# Multiplexed detection of serological cancer markers with plasmon-enhanced Raman spectro-immunoassay†
†Electronic supplementary information (ESI) available: Fig. S1–S7 and Table S1. See DOI: 10.1039/c5sc01054c



**DOI:** 10.1039/c5sc01054c

**Published:** 2015-04-28

**Authors:** Ming Li, Jeon Woong Kang, Saraswati Sukumar, Ramachandra Rao Dasari, Ishan Barman

**Affiliations:** a Department of Mechanical Engineering , Johns Hopkins University , Baltimore , Maryland 21218 , USA . Email: ibarman@jhu.edu; b Laser Biomedical Research Center , George R. Harrison Spectroscopy Laboratory , Massachusetts Institute of Technology , Cambridge , Massachusetts 02139 , USA . Email: liming0823@gmail.com; c Department of Oncology , Johns Hopkins University School of Medicine , Baltimore , Maryland 21287 , USA

## Abstract

Circulating biomarkers have emerged as promising non-invasive, real-time surrogates for cancer diagnosis, prognostication and monitoring of therapeutic response. Emerging data, however, suggest that single markers are inadequate in describing complex pathologic transformations. Architecting assays capable of parallel measurements of multiple biomarkers can help achieve the desired clinical sensitivity and specificity while conserving patient specimen and reducing turn-around time. Here we describe a plasmon-enhanced Raman spectroscopic assay featuring nanostructured biomolecular probes and spectroscopic imaging for multiplexed detection of disseminated breast cancer markers cancer antigen (CA) 15-3, CA 27-29 and cancer embryonic antigen (CEA). In the developed SERS assay, both the assay chip and surface-enhanced Raman spectroscopy (SERS) tags are functionalized with monoclonal antibodies against CA15-3, CA27-29 and CEA, respectively. Sequential addition of biomarkers and functionalized SERS tags onto the functionalized assay chip enable the specific recognition of these biomarkers through the antibody-antigen interactions, leading to a sandwich spectro-immunoassay. In addition to offering extensive multiplexing capability, our method provides higher sensitivity than conventional immunoassays and demonstrates exquisite specificity owing to selective formation of conjugated complexes and fingerprint spectra of the Raman reporter. We envision that clinical translation of this assay may further enable asymptomatic surveillance of cancer survivors and speedy assessment of treatment benefit through a simple blood test.

## Abstract 2

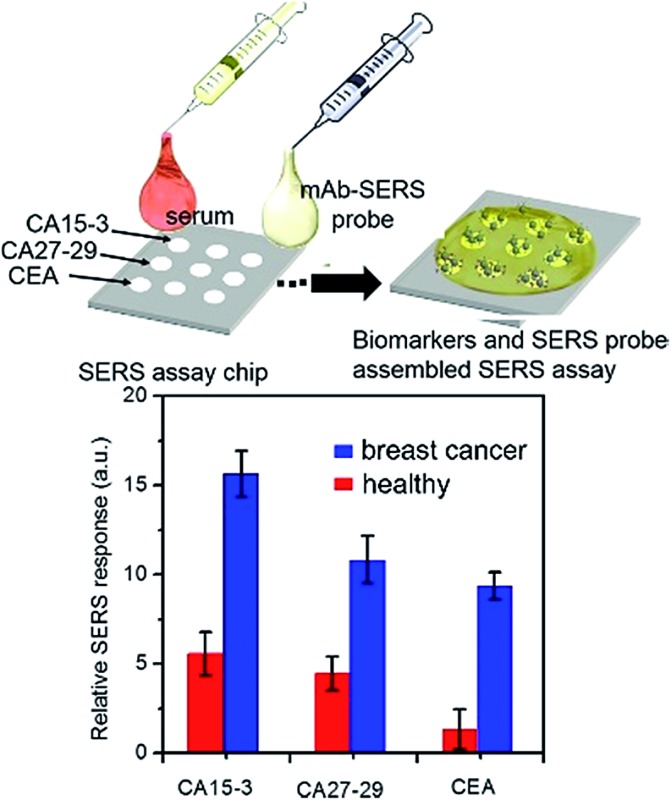
A plasmon-enhanced Raman spectroscopic assay has been developed for multiplexed detection of breast cancer markers—with high sensitivity and exquisite specificity, offering the potential of evaluating the breast cancer burden accurately.

## Introduction

Despite recent advances in the understanding of breast cancer progression and in the development of therapeutic modalities, breast cancer remains a global problem with a significant mortality rate and an equally substantial socio-economic burden.^1–4^ Our rudimentary knowledge of local recurrence and distant metastatic breast cancer is primarily responsible for the continued loss of lives. While local breast cancer responds very well to therapy and has a 5 year survival near 98%, the 5 year survival rate for metastatic breast cancer that involves distant organs drops to a dismal 24%.^5^ Extending life expectancies, therefore, requires sustained research in monitoring and managing recurrence and metastatic disease. Specifically, sensitive measurement of changes in tumor burden will assist the development of optimal treatment strategies for metastatic breast cancer. Moreover, early detection of recurrence prior to diagnosis by conventional modalities such as radiographic imaging will allow surveillance of asymptomatic cancer survivors.

In this milieu, there has been a burgeoning interest in circulating biomarkers owing to their potential for diagnosis, prognostication and monitoring response to systemic therapies in the neoadjuvant, adjuvant and metastatic settings.^6^ While promising data has recently been reported on circulating tumor cells and circulating tumor DNA,^7,8^ serum-based glycosylated tumor markers, notably cancer antigen 15-3 (CA15-3), CA27-29 and carcinoembryonic antigen (CEA), represent the most mature panel for monitoring patients with metastatic disease.^9–12^ These biomarkers are significantly overexpressed in stage IV breast cancer patients, which contain much higher concentrations than normal levels of <30 U mL^–1^, <38 U mL^–1^ and <10 ng mL^–1^ for CA15-3, CA27-29 and CEA, respectively.^9,13,14^ Despite being endorsed by American Society of Clinical Oncology, however, their utility has been limited by the sensitivity and specificity of the individual markers.^15^ To overcome this drawback, a shift in paradigm towards concomitant measurement of multiple markers has gained impetus.^16^ Yet, current diagnostic techniques, including enzyme-linked immunosorbent assay (ELISA), radioimmunometric assay and Western blot, do not provide the necessary multiplexing functionality and additionally often suffer from limited sensitivity and heavy interference from biological matrices.^17,18^ Given these limitations, a single blood-based test for these tumor antigens is still to be incorporated into a clinical laboratory assay.

Here we present a multiplex surface-enhanced Raman spectroscopy (SERS)-based assay for sensitive and specific detection of the tumor antigen panel. Our approach combines spectroscopic imaging with tailored SERS probes, where the signal enhancement arises from the proximity of the Raman reporter molecule to the intense localized plasmonic fields created by the nanostructured metals.^19–24^ The signal of this reporter transduces the presence (and concentration) of the tumor antigen at extremely low concentrations to a quantitative and reproducible spectral pattern. We designed a SERS chip that comprises pre-defined wells patterned in a quartz substrate. Each array is functionalized with monoclonal antibody (mAb) for different tumor antigens. Using a Raman microscope to scan the chip, the individual spectra are integrated into numerical algorithms for robust estimation of the expression levels. We show that this assay offers multiplexing capability in a single serum droplet (∼2 μL) while achieving a high sensitivity and molecular specificity. We further developed a wide-area, compact Raman spectroscopic scanner that can sample the chip in a small fraction of the time necessary for standard chemical imaging. Collectively, these findings underline the transformative potential of this assay for serum expression.

## Results and discussion

**Fig. 1:**
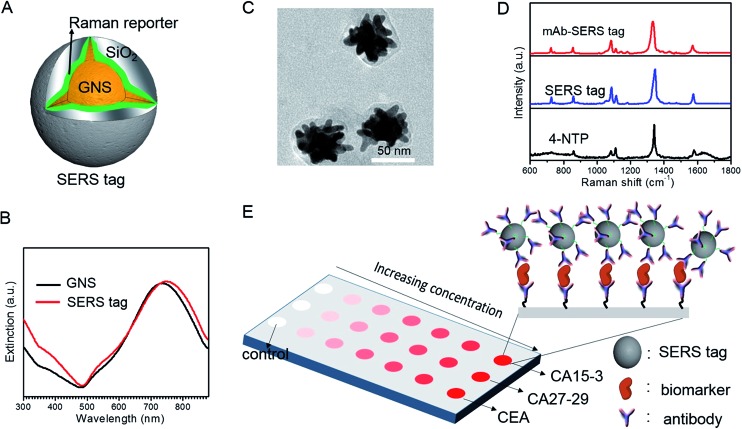
Multiplexing SERS assay using encoded gold nanostar (GNS) SERS probes. (A) Schematic structure of a SERS tag where a number of Raman reporter molecules, 4-nitrothiophenol (4-NTP), are sandwiched between GNS and thin silica layer. (B) Extinction spectra of bare GNS and SERS tags in aqueous solution, showing the *ca.* 14 nm red-shift in LSPR absorption maximum of SERS tags (748 nm) compared with the bare GNS (734 nm). (C) Representative TEM image of SERS tags. (D) Raman and SERS spectra of 4-NTP, SERS tag and CA15-3 mAb-modified SERS tag (SERS probe). (E) Schematic illustration of SERS assay for multiplex detection of biomarkers. Imaging is performed over a wide field of the wells in the SERS panel and spatial average of the SERS response is correlated to the levels of corresponding biomarkers. For each measurement, the control is used as the internal standard to calibrate the SERS response.

We employed gold nanostars (GNS) as the basis for designing SERS probes with substantive signal enhancement and exceptional multiplexing capability (Fig. 1A).^22,23^ By modulating the protrusion length and density as well as the core size, we optimally tuned the localized surface plasmon resonance (LSPR) of the GNS to 734 nm and observed that the thin silica coating caused a slight red-shift to 748 nm (Fig. 1B). The interplay between plasmonic enhancement and optical extinction causes the GNS with LSPR blue-shifted (off-resonant) from the 785 nm excitation wavelength to provide the maximum net amplification in the colloidal suspension.^22,25^ We embedded Raman reporter, 4-nitrothiophenol (4-NTP), on the GNS surface, which was then coated with a thin silica layer (∼5 nm thickness, Fig. 1C and S3†). The silica coating enables flexible surface functionalization rendering the desired molecular specificity and prevents the leaching of 4-NTP during the processing and assay operations. Next, we used standard amine coupling chemistry to graft antibodies (CA15-3 monoclonal antibody (mAb), CA27-29 mAb and CEA mAb) to carboxyl group-modified SERS tags (Fig. S1†).^23^Using the Raman microscope, we acquired spectra from 4-NTP, SERS tags and the mAb-modified SERS tags (SERS probes) for probe characterization (Fig. 1D). The acquisition confirmed that the signatures of the SERS tags and the CA15-3 targeted probes were identical to that of 4-NTP (Fig. 1D and Table S1†). Similar results were also observed for CA27-29 and CEA targeted probes (Fig. S2†).

Additionally, each well in the SERS chip was functionalized with carboxyl group and activated with the standard amine coupling chemistry, followed by conjugation with the respective antibodies (Fig. 1E and S1†). Here the mAb molecules immobilized on the quartz slide act as the capture probe and the mAb molecules on the SERS tag surface serve as the recognition moiety on the detection probes for the biomarkers. Bovine serum albumin was used as the surface blocking reagent to avoid nonspecific adsorption of extraneous species on the chip surface (Fig. S1†).^26^ The chip bound with biomarkers was then incubated in a solution containing SERS probes forming the sandwich assay configuration. After removal of the free SERS probes, the chip was subjected to spectral acquisition. We performed spectroscopic imaging, as opposed to single point measurements, to improve signal robustness through spatial averaging and to minimize sampling errors.

**Fig. 2:**
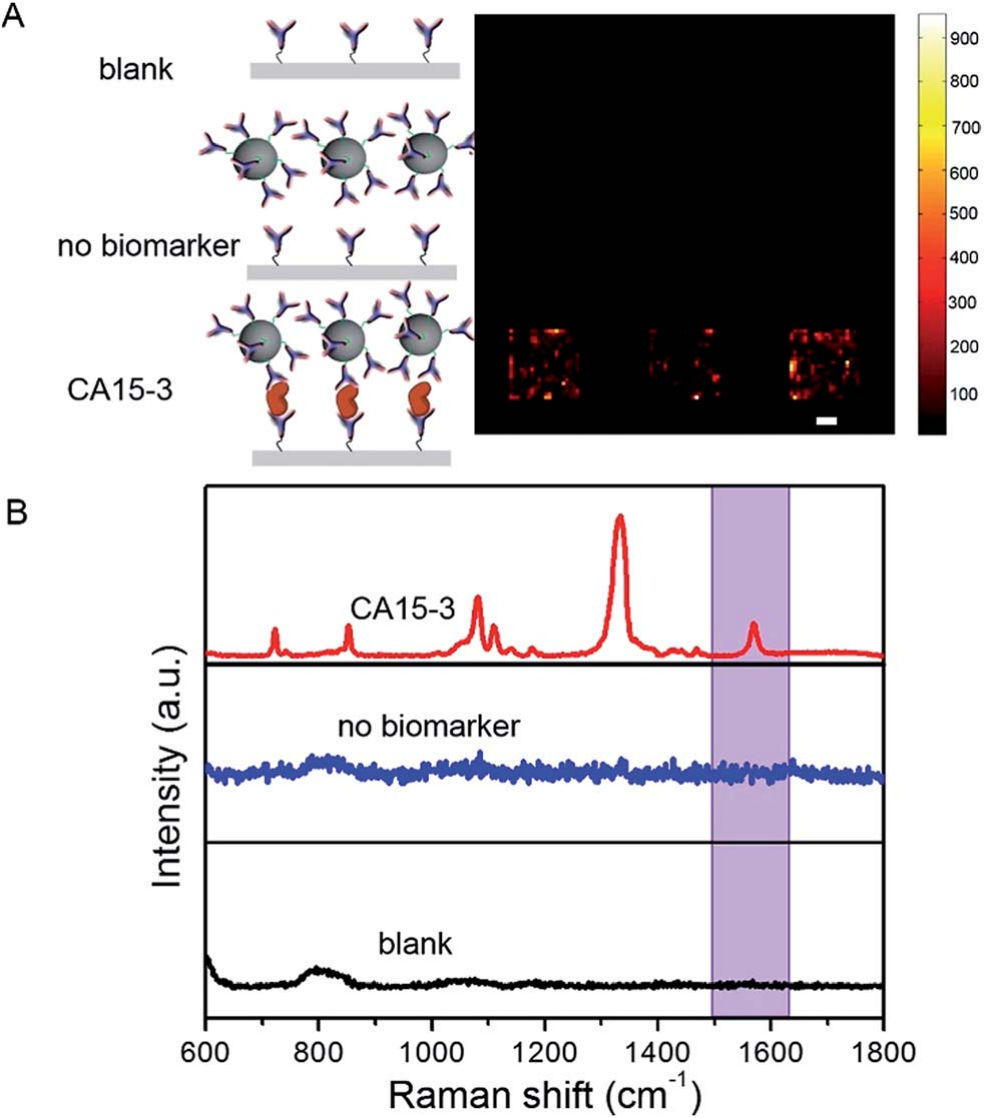
Proof-of-concept experiments for SERS assay of CA15-3 antigen. (A) SERS spectroscopic images and (B) Corresponding average SERS spectral response in the absence/presence of SERS tag and CA15-3 antigen. Blank indicates that the SERS measurement is directly performed on CA15-3 mAb-modified quartz substrate without addition of either CA15-3 SERS probe or CA15-3 antigen; no biomarker indicates SERS acquired from the SERS platform in the presence of CA15-3 SERS probe but in the absence of CA15-3 antigen. SERS responses represent acquisition intensities when 100 U mL^–1^ concentration of CA15-3 antigen is incorporated to complete the sandwich assay. All experiments are triply performed in parallel, and the relative SERS response with respect to the blank is used to generate the SERS image. Scale bar in (A) is 20 μm. Highlighted area (1500 cm^–1^ to 1630 cm^–1^) in (B) indicates the area surrounding the characteristic Raman peak (1570 cm^–1^) that is used for construction of SERS images in (A) and the ensuing analysis.

To determine the feasibility of the SERS chip for biomarker detection, we first performed proof-of-concept experiments in PBS buffer media (Fig. 2). The sandwich configuration, in the presence of 100 U mL^–1^ CA15-3, faithfully reproduces the signal of the Raman reporter. In contrast, the control experiments confirm that there was no observable signal in the “blank” as also when only the SERS probe was added. The latter can be attributed to the fact that the SERS probes are easily washed away when the sandwich configuration *via* the antibody-antigen binding is not formed. In order to display the SERS chip response, we constructed spectral images based on the integral area of the 1570 cm^–1^ Raman peak (Fig. 2A). We observed substantially brighter SERS images in the presence of CA15-3 antigen—the imaging equivalent of the single point spectral acquisition shown in Fig. 2B.

**Fig. 3:**
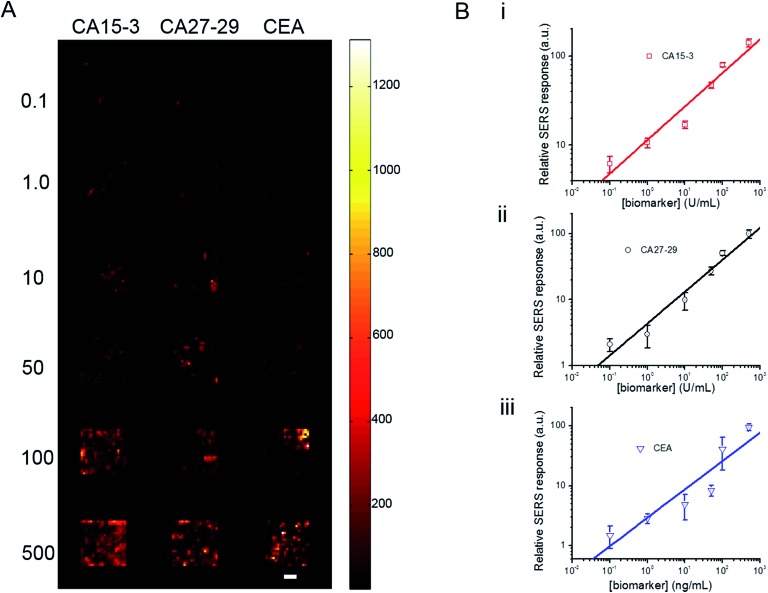
Spectroscopic images from the SERS assay for detection of CA15-3 antigen and concentration-dependent SERS response for CA15-3, CA27-29 and CEA antigens in PBS buffer solution. (A) Spectroscopic images from the SERS assays of CA15-3, CA27-29 and CEA antigens in buffer. Three capture probes against CA15-3, CA27-29 and CEA are immobilized onto the pre-defined wells patterned on a quartz slide, and the biomarkers (CA15-3, CA27-29 and CEA) of various concentrations are then applied. The labels on the left indicate the corresponding concentrations for each image. Scale bar in (A) is 20 μm. (B) Concentration-dependent relative SERS responses of (i) CA15-3, (ii) CA27-29 and (iii) CEA. In each assay, the ratio of the average SERS response over the examined region in the sandwich assay to that of the control experiment (blank) is used as the relative SERS response. The results are presented on the basis of parallel triplicate experiments.

To investigate its applicability as a quantitative assay, we next examined the SERS response upon varying the biomarker concentrations in the ranges encountered in clinical practice (Fig. 3A and B).^9,13,14^ Concentrations both lower and higher than the clinically relevant levels were also included to obtain a comprehensive assessment of the dynamic range. Specifically, six concentrations of CA15-3, CA27-29 and CEA were spotted on the SERS chip. The SERS response shows a consistent increase in intensity (brightness) due to more captured SERS probes per well with rising concentration for all three biomarkers. We also correlated the relative SERS response, in relation to the control, with the various biomarker concentrations (Fig. 3B). Substantive linearity was observed in the log–log calibration curve over the examined concentration ranges, 0.1 U mL^–1^ to 500 U mL^–1^ for CA15-3 (coefficient of determination *R* ^2^ = 0.94) and CA27-29 (*R* ^2^ = 0.95), and 0.1 ng mL^–1^ to 500 ng mL^–1^ for CEA (*R* ^2^ = 0.97).

**Fig. 4:**
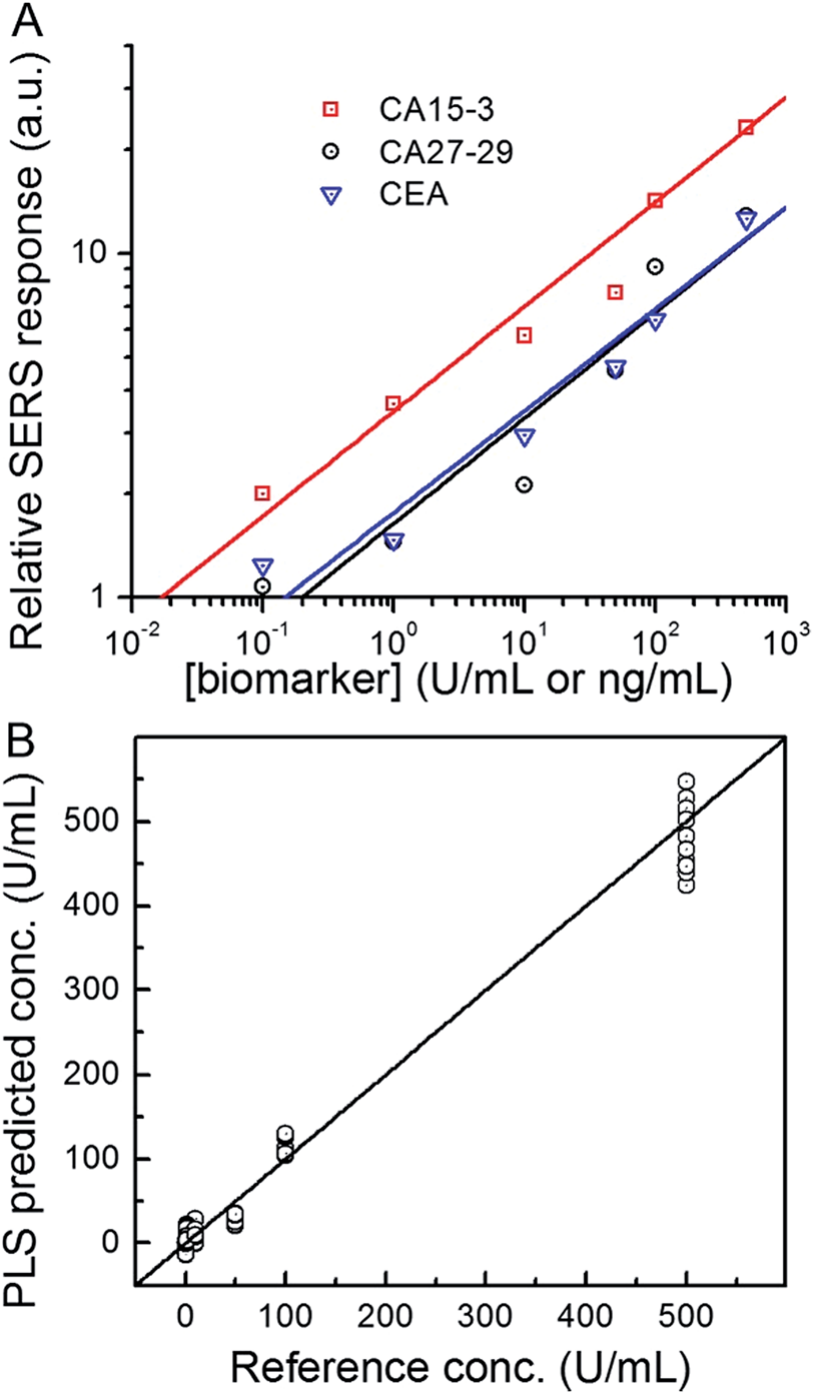
Concentration-dependent SERS assay of CA15-3, CA27-29 and CEA antigens in serum. (A) Relative SERS response as a function of the biomarker concentration. (B) Representative partial least squares (PLS) prediction results for CA27-29 quantification in serum. The solid line denotes *y* = *x* values. Samples were prepared by spiking the biomarkers in fetal bovine serum (0.1, 1.0, 10, 50, 100 and 500 U mL^–1^ for CA27-29).

Next, we used the SERS chip for biomarker detection in serum (Fig. 4A and S4†). The logarithm of the SERS responses increases linearly with the logarithm of biomarker concentrations investigated with *R* ^2^ values equal to 0.98, 0.90 and 0.99 for CA15-3, CA27-29 and CEA, respectively. We further analyzed the binding characteristics of the biomarkers by fitting the experimental data to Langmuir isotherms, which yielded dissociation constants of 95.9 U mL^–1^, 83.1 U mL^–1^ and 113.2 ng mL^–1^ for CA15-3, CA27-29 and CEA, respectively (Fig. S5†). Although the spectral intensity values are lower in sera than those obtained in buffer, the acquired profiles and the response curves highlight the molecular specificity *via* the lack of interference from the myriad endogenous constituents of the sera. Additionally, we used multivariate regression analysis for concentration prediction as it exploits the entire spectral information (rather than focusing on a single peak) and has the associated advantage of noise averaging across the spectrum. Leave-one-out cross-validation was performed using partial least squares (PLS) regression (Fig. 4B and S6†).^27^ Evidently, there is close agreement between the predicted and reference concentrations with *R* ^2^ values of 0.98, 0.99 and 0.99 for CA15-3, CA27-29 and CEA, respectively. Importantly, the limits of detection (LOD) were computed to be 0.99 U mL^–1^, 0.13 U mL^–1^ and 0.05 ng mL^–1^ for CA15-3, CA27-29 and CEA, respectively. These values are significantly smaller than the corresponding LOD values reported from the conventional methods, such as commercial ELISA kits (widely treated as the gold standard for proteomics assays): 5.0 U mL^–1^ for CA15-3, 3.8 U mL^–1^ for CA27-29 and 1.0 ng mL^–1^ for CEA.^28^ We note that the SERS responses shown in Fig. 3 and 4A are slightly different for each antigen, which may be attributed to the different antibody-antigen binding affinities.

**Fig. 5:**
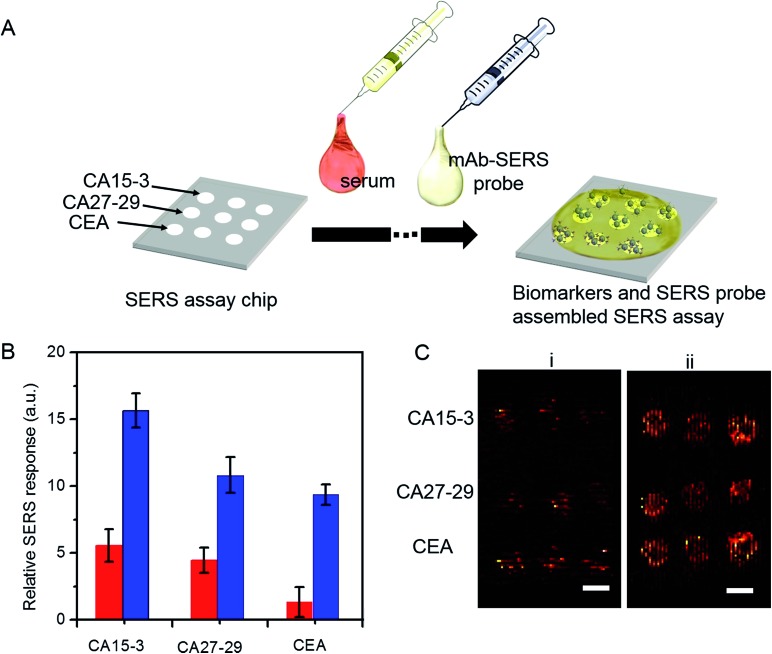
Multiplexing assays of serum samples with healthy and patient biomarker concentrations. (A) Schematic illustration of SERS assay chip for multiplexing assay of biomarkers at a single test. Briefly, the SERS assay panel is functionalized with CA15-3 mAb, CA27-29 mAb and CEA mAb in their respective defined regions. A drop of serum sample is deposited onto the panel and covers the entire region. After the incubation, a mixture of SERS probes functionalized with various mAb molecules are dropped. After vigorously washed with PBS buffer solution, the SERS assay panel is subject to the SERS assay. (B) Relative SERS response for healthy (red) and patient (blue) serum samples (for healthy sample, CA15-3:10 U mL^–1^, CA27-29: 30 U mL^–1^ and CEA: 1 ng mL^–1^; for patient sample, CA15-3: 150 U mL^–1^, CA27-29: 180 U mL^–1^ and CEA: 200 ng mL^–1^). The mean integral area over the examined region is divided by that from the blank to give the relative SERS response. Three independent experiments are performed in parallel for each type of serum sample. (C) SERS images acquired by Raman spectroscopic scanner. Images from spiked serum samples mimicking the concentrations observed in (i) healthy serum sample and (ii) patient serum sample. For both i and ii, the first row is for CA15-3, the second row for CA27-29 and the third row for CEA. Each experiment is triply performed in parallel. Scale bar in (C) is 1.5 mm.

A key advantage of our platform is its multiplexing ability. To test this feature, we architected a 3 × 3 array of sensing units with each row dedicated to measurement of a specific antigen and the three columns enabling triplicate measurements. A single drop of serum (∼2 μL) spiked with differing quantities of the three cancer antigens was pipetted to cover the whole chip, followed by sequential addition of the mAb-SERS probes (Fig. 5A). During the incubation period, the serological markers and mAb-SERS probes together form the sandwich assay configuration with the capture probes on the corresponding wells. Without any other pretreatment, we employed spectroscopic imaging on the chip to render direct and simultaneous readout of the tumor antigen concentrations. We examined two serum samples spiked with different concentrations of the antigens. The antigen concentrations in the first sample resembled the levels of a healthy individual whereas the concentrations in the second sample were consistent with observations in metastatic breast cancer patients (Fig. 5B). We observe that the first sample generates a weak, yet observable, SERS signal. In contrast, the SERS intensity from the second specimen exhibits a significantly larger response in each case. We also quantified the antigen concentrations on the basis of the acquired spectra and the previously formulated PLS calibration models. The predicted values show excellent agreement with the reference concentrations with relative errors of prediction of 10.4%, 3.0% and 6.0% for CA15-3, CA27-29 and CEA, respectively (Fig. S7†). Relative standard deviations were calculated to be 13.5% (CA15-3), 4.0% (CA27-29) and 8.4% (CEA), which are deemed to be clinically acceptable. Furthermore, our result demonstrates the low interference from other biomarkers, *i.e.* robustness to cross-reactivity (stemming from the antibody-antigen affinity), despite the high biomarker concentrations in the serum specimen representative of the patient sample.

Finally, we assessed the feasibility of higher throughput SERS measurements using a simpler, portable imaging system. We developed a wide-field compact scanning setup to address the limited sampling area and the substantive costs of a Raman microscope. Consisting of a laser diode and an air-cooled CCD imager, the flatbed scanner offered a large field of view (100 cm^2^). Despite the system's relatively lower detection sensitivity, we observed that the acquired images still allow clear differentiation between the two spiked serum samples (Fig. 5C) with the sample mimicking breast cancer patient antigen levels exhibiting markedly higher SERS response. The wide field of view enables direct visualization of the entire 3 × 3 panel with a 5-fold reduction in acquisition time. Coupled with the facile readout of the flatbed scanner, the SERS chip promises a highly sensitive and specific tool that can be further refined to create an inexpensive, point-of-care platform.

## Conclusions

Rapid, multiplexed tumor antigen analysis could improve early disease diagnosis and therapy response monitoring. We have developed a new liquid biopsy tool for multiplex detection of a panel of circulating tumor antigens based on plasmon-enhanced spectroscopic imaging. The structured nanoprobes realize substantive signal amplification while the attached Raman reporter independently tailors the spectral response. Moreover, the nanoprobes have the excitation and emission spectral signatures in the clear near infrared window and are designed to suppress both intimate contact (Raman) and through-space (fluorescence) enhancement of endogenous markers. We demonstrate that the proposed SERS platform shows high detection sensitivity. The strong proof-of-concept data generated here provides the needed momentum to pursue clinical feasibility studies for metastatic cancer diagnosis and longitudinal monitoring of chemotherapy response in breast cancer patients. This approach can also, in principle, be extended to detect circulating genetic and epigenetic markers such as microRNA and hypermethylated tumor DNA by substituting only the recognition moiety in the capture and detection probes. Finally, while we have employed breast cancer as the paradigm, this approach is generally applicable to other diseases including prostate, lung and colorectal adenocarcinomas, where pathologic conditions are complexly manifest in patterns of multiple biomarker expression levels.

## Experimental section

### Materials and chemicals

Gold chloride hydrate (HAuCl_4_·*x*H_2_O, 99.999% trace metals basis), trisodium citrate dihydrate (HOC(COONa) (CH_2_COONa)_2_·2H_2_O, ≥99%), poly(vinylpyrrolidone) (PVP, (C_6_H_9_NO)_*n*_, MW-10 kg mol^–1^), sodium borohydride (≥99%), *N*,*N*-dimethyformamide (DMF, anhydrous 99.8%), sodium hydroxide (pellets, 99.99% trace metals basis), (3-aminopropyl) trimethoxysilane (APTMS, 97%), sodium silicate (Na_2_O(SiO_2_)_*x*_·*x*H_2_O, reagent grade), 4-nitrothiophenol (4-NTP, technical grade 80%), *N*-hydroxysuccinimide (NHS, 98%), *N*-ethyl-*N*′-(3-dimethylaminopropyl)carbodiimide (EDC, ≥97.0%) and bovine serum albumin (BSA) were purchased from Sigma-Aldrich (St. Louis, MO). (3-Triethoxysilyl) propylsuccinic anhydride (TEPSA, C_13_H_25_O_6_Si, >95%) was purchased from Gelest, Inc. (Morrisville, PA). Cancer antigen 15-3 monoclonal antibody (CA15-3 mAb), CA15-3 antigen, CA27-29 antigen, carcinoembryonic antigen (CEA) and CEA monoclonal antibody (CEA mAb) were obtained from MyBioSource (San Diego, CA). Mouse anti-cancer CA27-29 monoclonal antibody (CA27-29 mAb) was purchased from Creative Diagnostics (Shirley, NY). Quartz cover slips (25.4 × 25.4 × 0.15–0.25 mm) were purchased from Alfa Aesar (Ward Hill, MA) for use as the SERS measurement substrate. Phosphate buffered saline (10× PBS) solution was purchased from OmniPur (Billerica, MA) and fetal bovine serum (FBS, BenchMark™) was acquired from Gemini Bio-Products (West Sacramento, CA). All other reagents or solvents were obtained from VWR (Radnor, PA) and used as received.

### SERS tag synthesis

SERS tags were synthesized according to our previously reported method with a slight modification.^22–24^ Briefly, gold nanostar (GNS) nanoparticles with the LSPR band maximum of 734 nm in aqueous solution were synthesized by employing the gold seed-mediated method.^22–24^ The GNS nanoparticles were dispersed into deionized water with a concentration of 1.7 pM for further use. To prepare the SERS tag, a freshly prepared solution of Raman reporter (4-NTP, 10 μM) was added dropwise to 15 mL GNS colloid while subject to rapid magnetic stirring. Stirring was continued for another 30 min before adding 10 μL of freshly prepared APTMS ethanolic solution (50 mM). After stirring for another 30 min, the pH value of reaction solution was adjusted to 9–10 by addition of NaOH aqueous solution. Following this, 200 μL of freshly prepared 0.54 wt% sodium silicate solution was added slowly, and then stirred for one day. 5 mL anhydrous ethanol was subsequently added to generate a condensed silica layer. The reaction solution was kept standing for one more day, centrifuged and washed with anhydrous ethanol and deionized water, respectively. Finally, the solid was dispersed into 0.5 mL 1× PBS buffer solution for further use.

### Antibody conjugation to quartz chip and SERS tags

#### Assay panel functionalization

Our underlying principle here is that by associating a set of antibodies with a particular row, a combinatorial utilization of the same nanoparticle-surface species with multiple antibodies can be implemented. To make the SERS assay panel, quartz slides were used and cleaned by subsequent sonication in ethanol and water. To pre-define the functional assay regions for different biomarkers, the Parafilm was bonded onto the cleaned quartz chip with punched wells (3 rows × 7 columns). In our assay, each row was defined for one type of biomarker. All of the following operations were carried out in a home-built humid chamber. Antibodies were immobilized onto the panel with pre-defined patterns through standard amine coupling chemistry. First, all wells were incubated over-night in an ethanol solution containing 100 mM TEPSA and then sequentially washed with ethanol and 1× PBS buffer solution to achieve a carboxyl group-modified surface. The resulting carboxyl-terminated quartz array panel was activated by immersion in a PBS buffer solution containing 50 mM NHS and 200 mM EDC. After washing with the PBS buffer solution to remove excess NHS and EDC, the panel was incubated overnight in the buffer solution of 100 μg mL^–1^ CA15-3 mAb (CA27-29 mAb or CEA mAb). The non-specifically bound antibodies were washed away with the 1× PBS buffer.

To achieve high assay specificity, it is crucial to minimize the non-specific biomarker adsorption. In this work, we used BSA as the surface blocking reagent because of its excellent stability and biocompatibility.^25^ The antibody-immobilized patterned panel was spotted and then incubated for 2 hours in a 1× PBS buffer solution of 1 mg mL^–1^ BSA, followed by rinsing with 1× PBS buffer solution. Next, the resultant assay was kept in the humid chamber for further SERS assembly.

### Antibody-conjugated SERS tags

The antibody-SERS tag conjugates (SERS probes) were synthesized as detailed elsewhere in the literature.^23^ First, the SERS tags with carboxyl groups were prepared by incubating 200 μL SERS tags overnight in a 0.12 M TEPSA buffer solution. The carboxyl group-modified SERS tags were washed twice with a 1× PBS buffer solution, and then dispersed into a 1× PBS solution contained 50 mM NHS and 200 mM EDC to activate the carboxyl terminal group. After the 2 hour incubation period, 100 μg mL^–1^ CA15-3 mAb (CA27-29 mAb or CEA mAb) was added onto the activated SERS tags in PBS buffer solution, and then incubated overnight. Unbound mAb residues were removed by centrifugation at 4000 rpm and subsequent washing with 1× PBS buffer solution at least three times. The resultant SERS probes were re-dispersed into 0.5 mL 1× PBS buffer solution for further use.

### SERS assay of biomarkers

We performed SERS assay experiments in two different matrices, namely, in 1× PBS buffer solution and in sera.

#### SERS assay in buffer

Various amounts of the biomarkers (CA15-3, CA27-29 or CEA) were spiked into 1× PBS buffer solution to achieve a range of biomarker concentrations (0.1, 1.0, 10, 50, 100 and 500 U mL^–1^ for CA15-3 and CA27-29 antigens, 0.1, 1.0, 10, 50, 100 and 500 ng mL^–1^ for CEA antigen). These concentrations are selected as it spans the clinically relevant range from that typically encountered in healthy individuals to patients with advanced breast cancer.^9,13,14^ 2 μL of each biomarker solutions with various concentrations was spotted onto the corresponding pre-defined pattern (*i.e.*, matrix arrangement of wells) on the SERS assay panel, and incubated for one hour. Next, the panel was carefully washed with 1× PBS buffer solution to remove any traces of unbound biomarkers. Subsequently, 2 μL of SERS probes were spotted on the corresponding patterns and incubated for one more hour, followed by vigorous rinsing with 1× PBS buffer solution. Finally, the sandwich assay was subjected to the SERS measurements.

#### SERS assay in serum

Similarly, the biomarker serum solutions at the aforementioned range of CA15-3, CA27-29 or CEA concentrations were prepared by spiking various amounts of as-received biomarkers into FBS serum. The lack of pre-existing biomarkers in FBS serum precluded any potential interference. SERS assays of biomarkers in sera were prepared following a similar procedure to that outlined above for the buffer solution.

### SERS measurements

SERS measurements were performed using a home-built, confocal, inverted Raman microscope. A Ti:Sapphire laser of 785 nm wavelength (3900S, Spectra-Physics) was used as the excitation source and a 1.2 NA, 60× water immersion objective lens (Olympus UPLASPO60XWIR) was used to focus the laser light to and collect the Raman-scattered light from the assay, as detailed in our previous work.^29^ The backscattered light was collected by a 50 μm multimode fiber (Thorlabs M14L01), delivered to a spectrograph (Holospec f/1.8i, Kaiser Optical Systems) and the dispersed light was finally detected by a TE-cooled, back-illuminated, deep depletion CCD (PIXIS:100BR eXcelon, Princeton Instruments). The SERS microscopic images were obtained using dual-axis galvo mirrors (CT-6210, Cambridge Technology). The SERS response (RSERS) at 1570 cm^–1^, characteristic of the Raman reporter (4-NTP), was computed by considering the integral of the area under the curve in the range of 1500 cm^–1^ to 1630 cm^–1^ and was used to construct the SERS images. The spatial average (*R*_SERS_) over the scanning region was used to calculate the SERS response in order to improve prediction robustness, given by:1
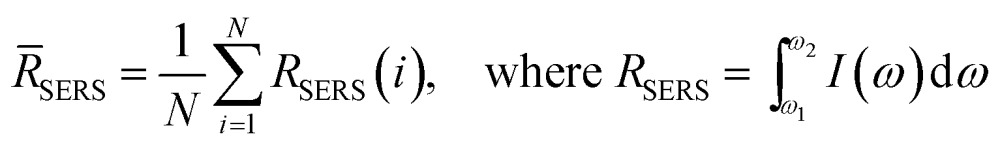
where *N* is the number (20 × 20) of spectra obtained over the scanning region, *ω* is the Raman shift in the integral range (1500 cm^–1^ to 1630 cm^–1^), and *I*(*ω*) is the Raman peak intensity at Raman shift, *ω*. All spectral measurements were obtained with an exposure time of 0.5 s at 4 mW laser power on the sample, unless otherwise noted.

Furthermore, in order to investigate the feasibility of high throughput, low-cost measurements, a flatbed Raman spectroscopic scanning system was constructed. Updated in design from our diffuse reflectance and autofluorescence scanner reported previously,^30^ wide area spectroscopic imaging capability is achieved by mechanically scanning the sample on top of inverted Raman imaging system with a quartz substrate. Spectral recording time was 100 ms per pixel. Here, a 785 nm compact solid-state laser is used as the excitation source and the collected light is recorded on a portable spectrometer.

### Characterization

Extinction spectra for the GNS and SERS tags were recorded on a Shimadzu UV-2401 spectrometer. Transmission electron micrographs (TEM) were acquired using the FEI Tecnai G2 Spirit TWIN transmission electron microscope at an accelerating voltage of 120 kV. The sample was dropped onto ultrathin Formvar-coated 200-mesh copper grids (Ted Pella, Inc.) and left to dry in air.

### Data analysis

To evaluate the efficacy of the present assay for quantitative concentration measurements, we performed partial least squares (PLS) regression analysis. Specifically, PLS calibration models were tested using the leave-one-sample-out cross-validation procedure for each biomarker. In this routine, one concentration is left out when developing the calibration model and the resultant model is used to predict concentrations of the left out concentration spectra.^30^ This procedure is repeated until all concentrations are left out and each of the concentrations has been predicted. In particular, the calibration models are developed using 50 spectra (5 concentrations with 10 spectra per concentration for each biomarker), and the predictions are performed on the remaining 10 spectra (1 concentration). Furthermore, the limit of detection (LOD) of the developed SERS assay is calculated from the best fit line obtained between the predicted concentrations and reference concentrations according to the IUPAC definition:^31^
2
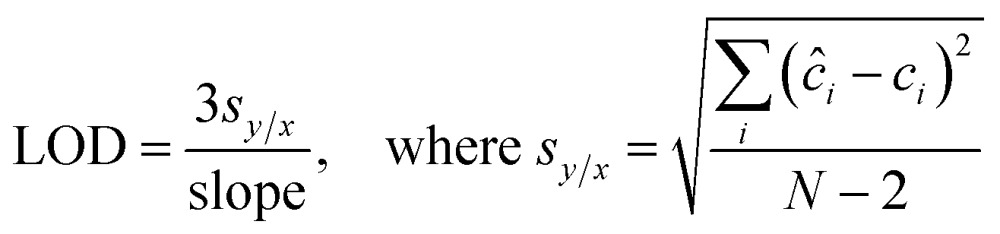
where *s*_*y*/*x*_ is the standard deviation of the residuals and is a measure of the average deviation of the prediction values from the regression line, *N* is the number of spectra in the dataset, *c*_*i*_ indicate the reference concentrations and *ĉ*_*i*_ the predicted concentrations.

We performed the similar PLS analysis for the spiked sera samples mimicking the concentrations of a healthy individual and a patient with advanced metastatic breast cancer to examine the accuracy and precision of quantitative measurements. Relative error of prediction (REP) and relative standard deviation (RSD) were calculated, which correlate directly with the accuracy and precision of SERS assay respectively. REP is calculated by the following equation:^30^
3
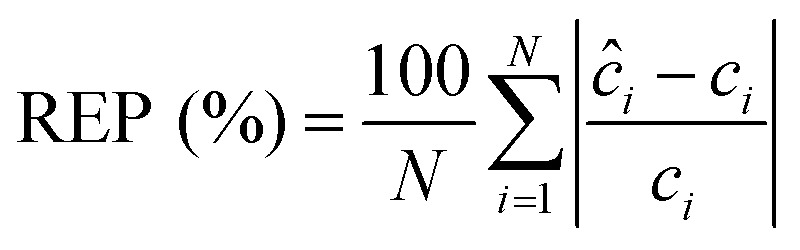



The RSD of predicted concentrations is given by:^30^
4
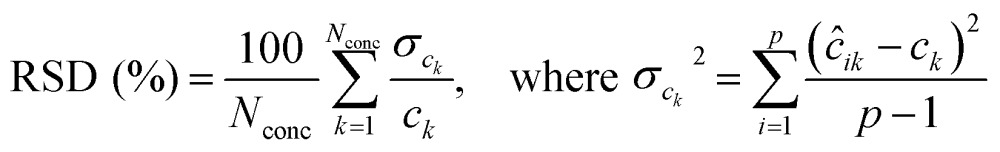
where *N*_conc_ is the number of distinct concentrations in the dataset, *p* is the number of spectra per concentration and *σ*_*c*_*k*__ is the standard deviation obtained at concentration *c*_*k*_.
