# Supplementary material for: Multiplexed detection of serological cancer markers with plasmon-enhanced Raman spectro-immunoassay
Source: Chem Sci. 2015 Apr 28;6(7):3906–14. doi: 10.1039/c5sc01054c (PMC4577055; doi:10.1039/c5sc01054c)
Supplement: Supplementary file 1 [file SC-006-C5SC01054C-s001.pdf]

Electronic Supplementary Material (ESI) for Chemical Science.
This journal is © The Royal Society of Chemistry 2015

Electronic Supplementary Information

Multiplexed detection of serological cancer markers with plasmon-enhanced Raman spectro-immunoassay

Ming Li, [a,b,*] *Jeon Woong Kang,* [b] *Saraswati Sukumar,* [c] *Ramachandra Rao Dasari,* [b] *and Ishan Barman* [a,c*]

[a]Department of Mechanical Engineering, Johns Hopkins University, Baltimore, Maryland 21218, United States

[b]Laser Biomedical Research Center, George R. Harrison Spectroscopy Laboratory, Massachusetts Institute of Technology, Cambridge, Massachusetts 02139, United States

[c]Department of Oncology, Johns Hopkins University School of Medicine, Baltimore, Maryland 21287, United States

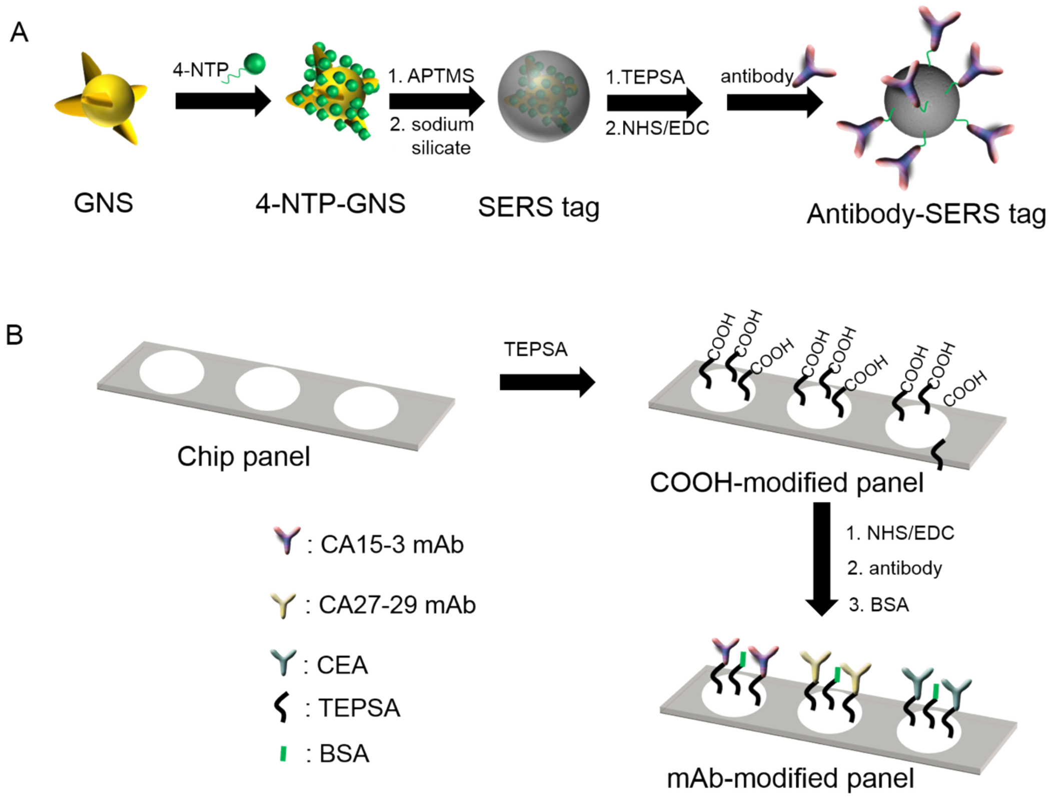

Figure S1. Schematic illustrations of SERS tag preparation and antibody functionalization on SERS tags and chip panel. (A) SERS tag synthesis and its conjugation onto antibodies (CA15-3 mAb, CA27-29 mAb and CEA mAb). (B) SERS assay panel modification with functional antibodies.

Table S1. Peak assignments of Raman and SERS spectra of 4-NTP and SERS tags[1,2]

Raman shift (cm^{-1})	Vibrational assignment
1574	Stretching vibration of phenyl ring
1333	Stretching vibration of N-O
1107	Bending vibration of C-H
1084	Stretching vibration of C-S
855	Wagging vibration of C-H
725	Wagging vibrations of C-H, C-S and C-C

1. S. Hong and X. Li, *J. Nanomater.,* 2013, **49**, 790323.
2. Z. Y. Bao, D. Y. Lei, R. Jiang, X. Liu, J. Dai, J. Wang, H. L. Chan and Y. H. Tsang, *Nanoscale,* 2014, **6**, 9063-9070.

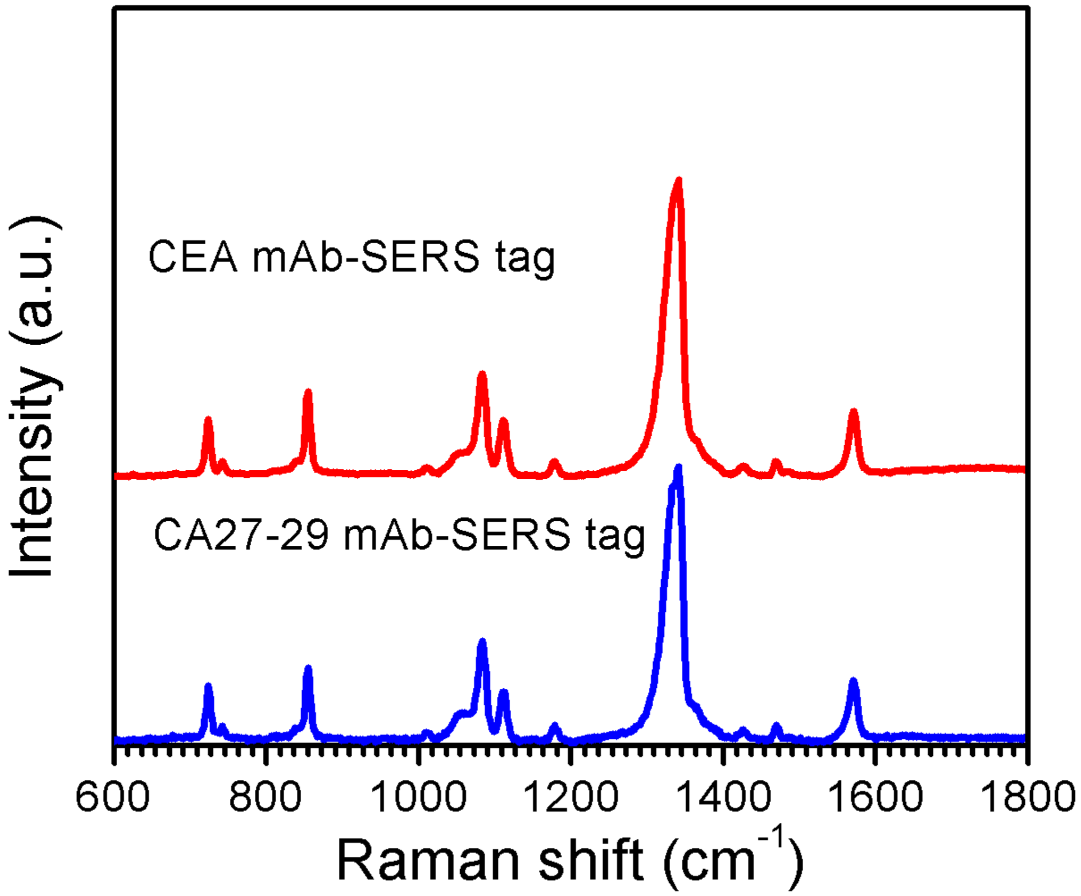

Figure S2. SERS spectra of CA27-29 mAb modified SERS tags and CEA mAb modified SERS tags.

Figure S3. TEM images of as-made GNS nanoparticles and SERS tags.

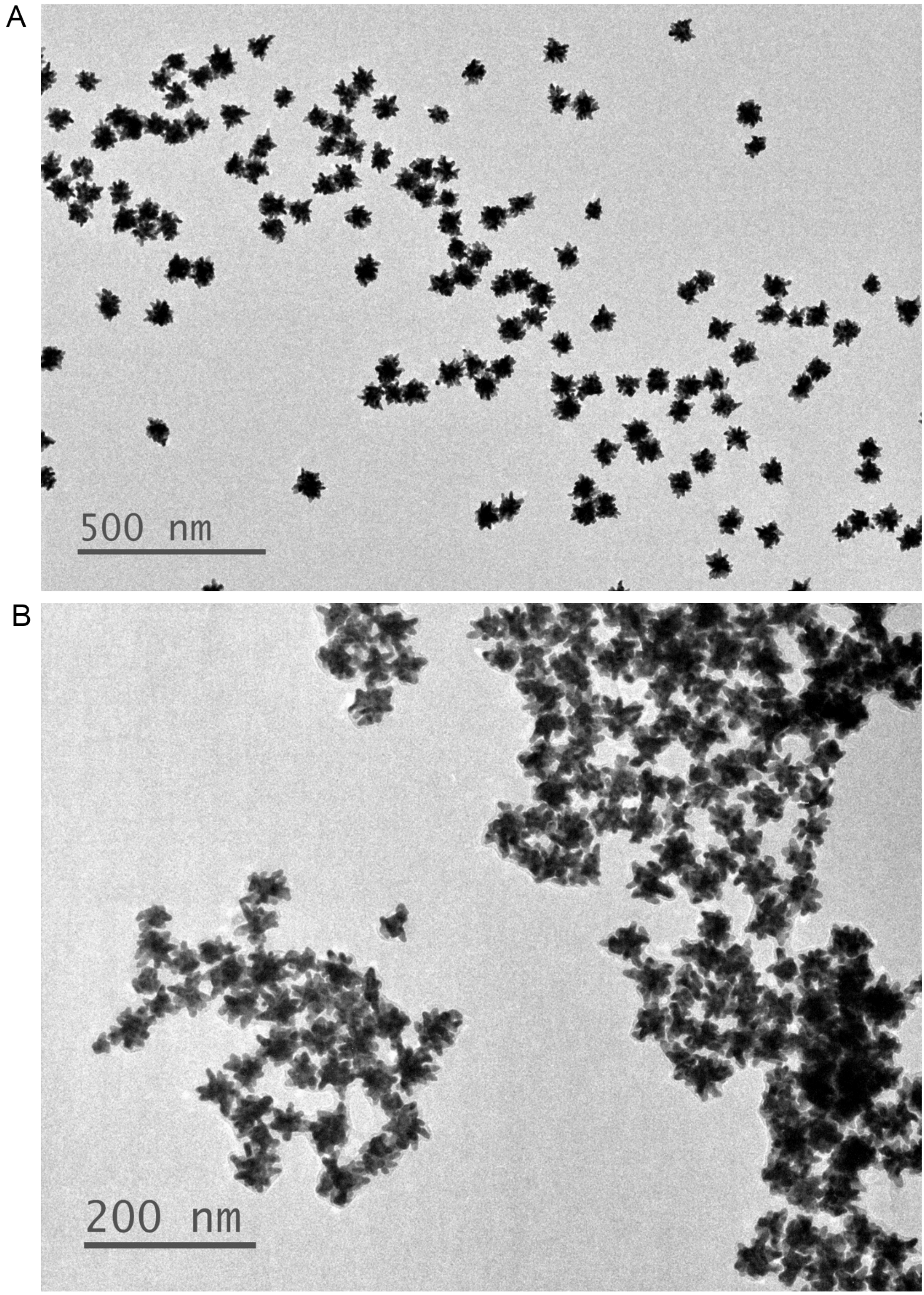

Figure S3. TEM images of as-made GNS nanoparticles and SERS tags.

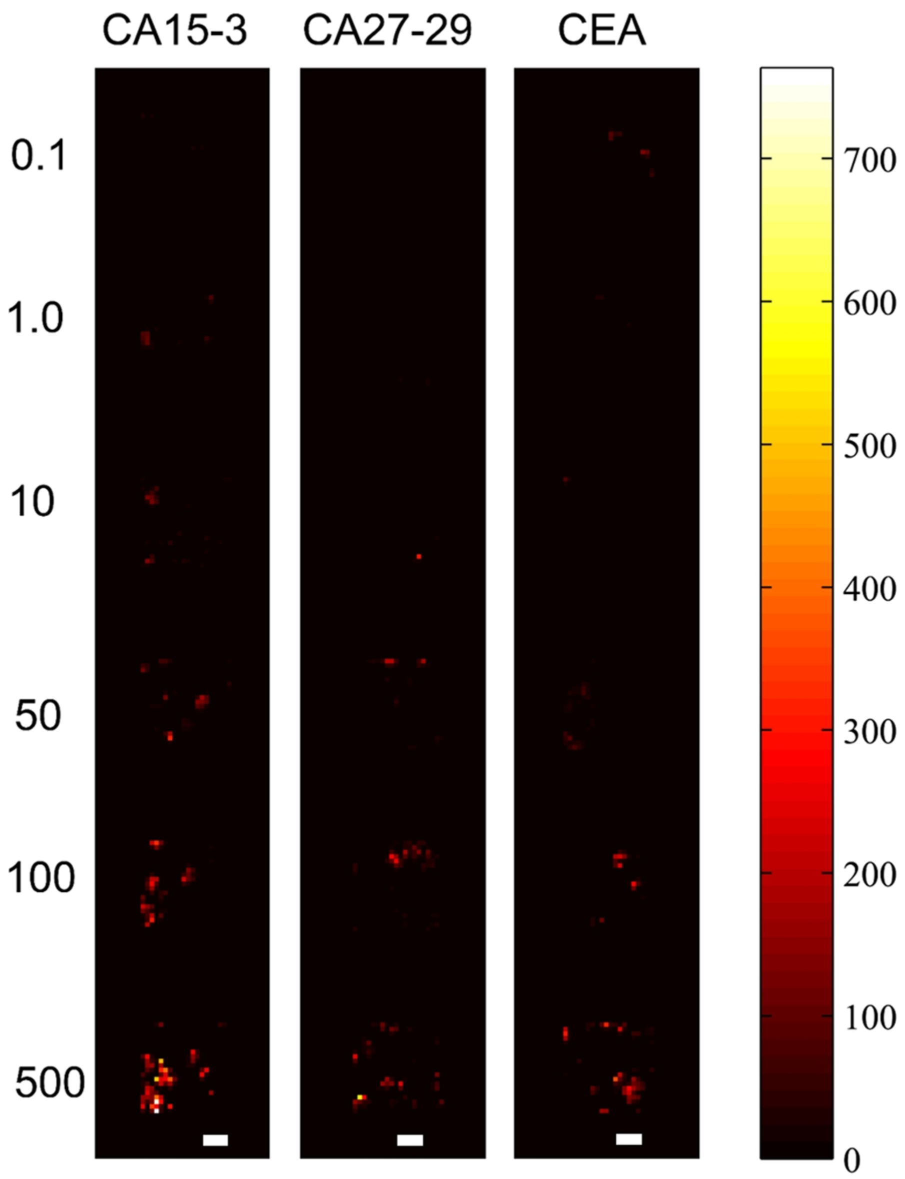

Figure S4. Concentration-dependent SERS assays of CA15-3, CA27-29 and CEA in serum. The concentrations are 0.1, 1.0, 10, 50, 100 and 500 U/mL for CA15-3 and CA27-29, respectively, while the concentrations are 0.1, 1.0, 10, 50, 100 and 500 ng/mL for CEA. Corresponding concentration for each image is shown in the left. Scale bar is 20 μm.

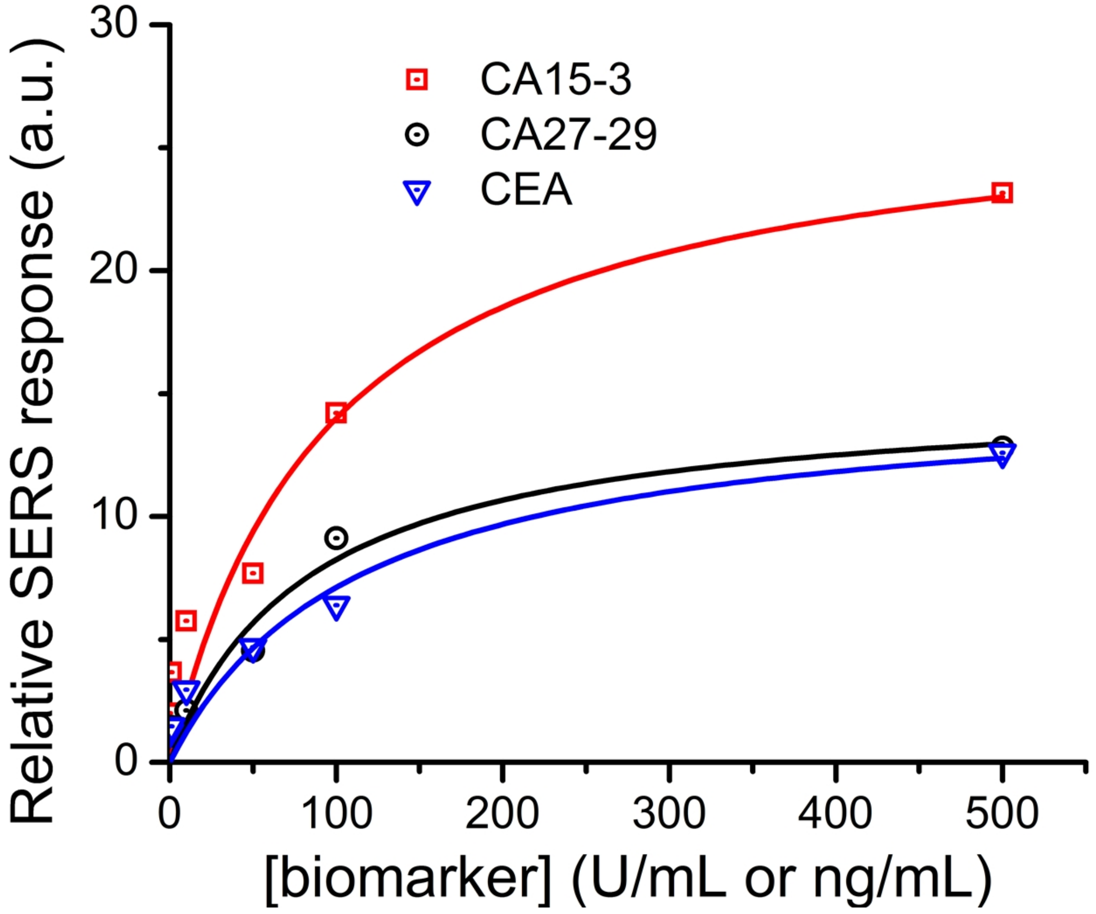

Figure S5. Concentration-dependent SERS assay of CA15-3, CA27-29 and CEA in serum. Fittings of curves are performed using Langmuir isotherms:

$$y = y_0 \bullet \frac{x}{k_d + x}$$

where y is relative SERS response, y_0 is a constant, x is the biomarker concentration, and k_d is the dissociation constant. Thus, we obtain the dissociation constants in sera: 95.9 U/mL for CA15-3, 83.1 U/mL for CA27-29, and 113.2 ng/mL for CEA, respectively.

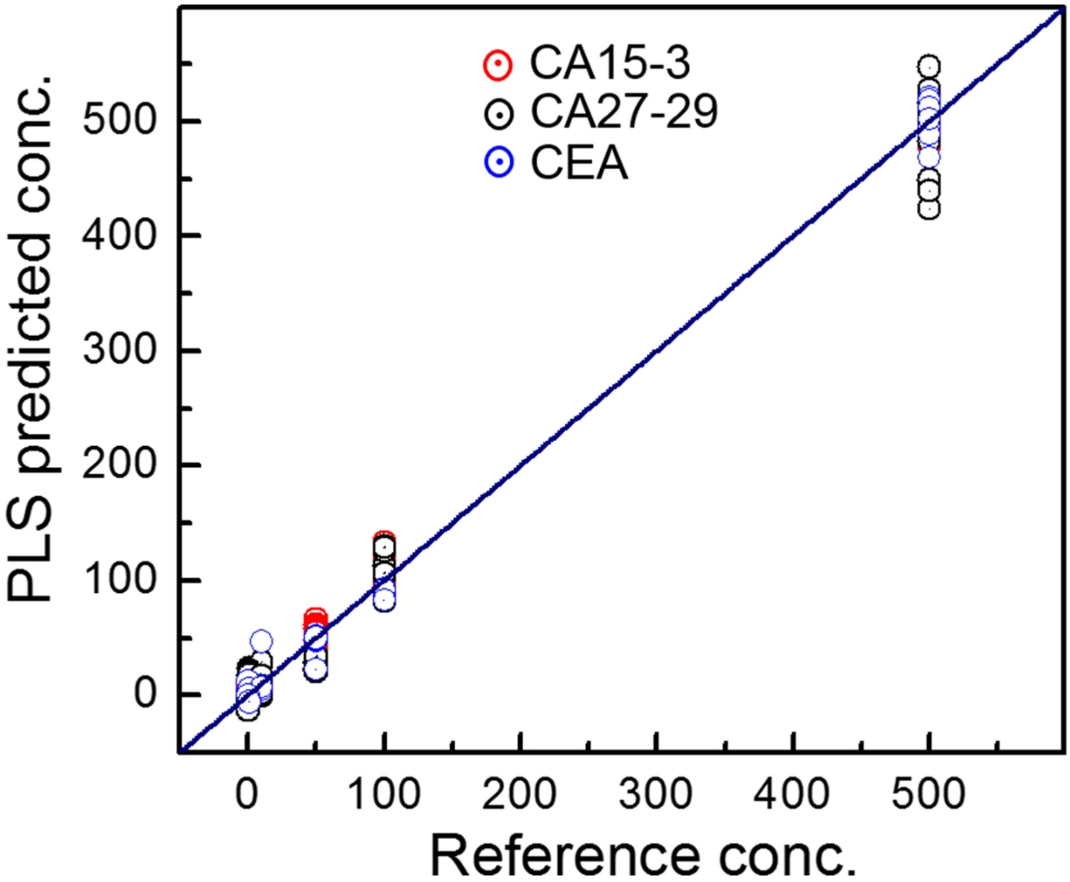

Figure S6. PLS regression analysis results for CA15-3, CA27-29 and CEA. The solid line denotes $y=x$ values. Samples were prepared by spiking the biomarkers in FBS (0.1, 1.0, 10, 50, 100 and 500 U/mL for CA15-3 and CA27-29, and 0.1, 1.0, 10, 50, 100 and 500 ng/mL for CEA).

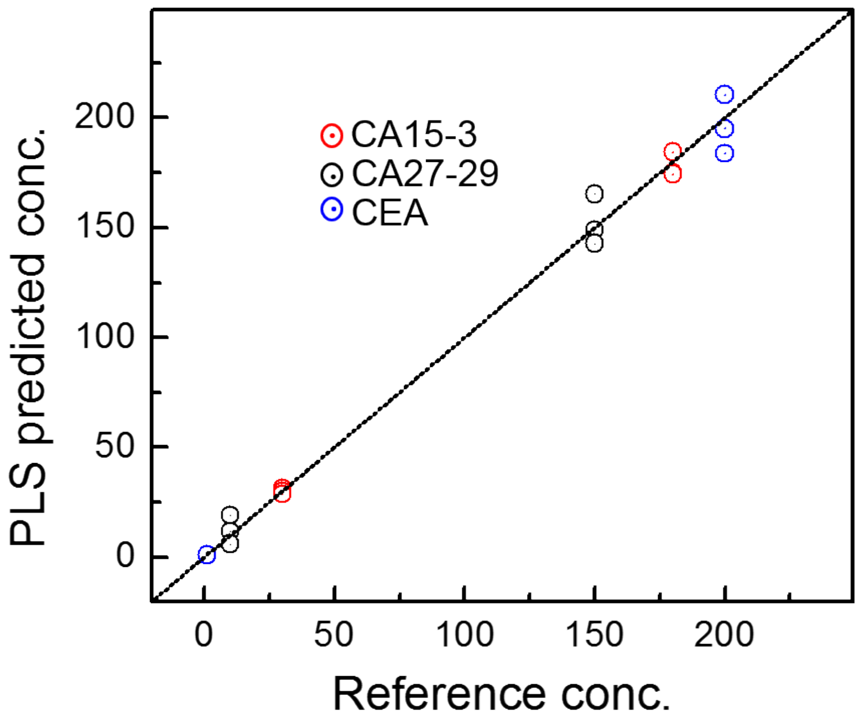

Biomarker	REP	RSD	R^2
CA15-3	10.4%	13.5%	0.98
CA27-29	3.0%	4.0%	0.99
CEA	6.0%	8.4%	0.99

Figure S7. PLS regression analysis of serum samples with healthy concentrations and patient biomarker concentrations. Table lists the resultant parameters from the PLS regression.
